# Efficient multiplex genome editing by CRISPR/Cas9 in common wheat

**DOI:** 10.1111/pbi.13508

**Published:** 2020-11-30

**Authors:** Jihu Li, Shujuan Zhang, Rongzhi Zhang, Jie Gao, Yiping Qi, Guoqi Song, Wei Li, Yulian Li, Genying Li

**Affiliations:** ^1^ Crop Research Institute Shandong Academy of Agricultural Sciences Jinan China; ^2^ Ministry of Agriculture Key Laboratory of Wheat Biology and Genetic Improvement on North Yellow and Huai River Valley Jinan China; ^3^ National Engineering Laboratory for Wheat and Maize Jinan Shandong China; ^4^ Department of Plant Science and Landscape Architecture University of Maryland College Park MD USA; ^5^ Institute for Bioscience and Biotechnology Research University of Maryland Rockville MD USA

**Keywords:** multiplex genome editing, CRISPR/Cas9, common wheat, ribozyme

Common wheat has a large genome with three subgenomes (A, B and D), making it challenging to create mutations at multiple genomic sites simultaneously. The CRISPR/Cas9 system offers a game‐changing tool for editing crop genomes (Chen *et al*., 2019). Three main strategies have been developed to produce multiple single‐guide RNAs (sgRNAs), including the conventional multiplex system with tandem repeats of separate U3 or U6 promoters (TRSP), the tRNA‐processing system (Xie *et al*., 2015) and the ribozyme‐processing system (Gao and Zhao, 2014). Although CRISPR/Cas9‐mediated genome editing was previously achieved by biolistic (Wang *et al*., 2014, 2018) and *Agrobacterium* transformation (Zhang *et al*., 2019a), a most efficient CRISPR/Cas9 system for multiplex editing in wheat remains elusive. To address this important question, we designed three multiplex editing constructs corresponding to these three systems, based on the pBUE411 vector (Figure 1a). For the TRSP system, wheat Pol III promoters, TaU3, TaU6.3 and TaU6.1 (Zhang *et al*., 2019a), were used to drive sgRNA expression independently. For the tRNA system, a TaU3 promoter was also used to express the tRNA‐sgRNA cassettes in a single transcript unit. For the ribozyme system, a Pol II promoter, Cestrum yellow leaf curling virus (CmYLCV) promoter (Cermak *et al*., 2017), was employed for expressing hammerhead ribozyme (HH)–sgRNA–hepatitis delta virus (HDV) ribozyme cassettes in a single transcript unit. A longer sgRNA scaffold was applied in three vectors to optimize the sgRNA structure (Dang *et al*., 2015). Wheat codon‐optimized Cas9 was driven under a maize (*Zea mays*) ubiquitin promoter (Ubip). Three genes, *TaDA1*, *TaPDS* and *TaNCED1*, were selected for simultaneous editing. The sgRNA for *TaDA1* could target its homoeologous genes on A and B chromosomes, while the sgRNAs for *TaPDS* and *TaNCED1* were designed to target all three homoeologous genes, respectively (Zhang *et al*., 2019b). In total, three sgRNAs could target 8 genomic sites in common wheat (Figure 1b). The sgRNA cassettes in the vectors were all arranged in the same order for close comparison. These T‐DNA vectors were introduced into hexaploid wheat Fielder via *Agrobacterium tumefaciens*‐mediated transformation.

**Figure 1:**
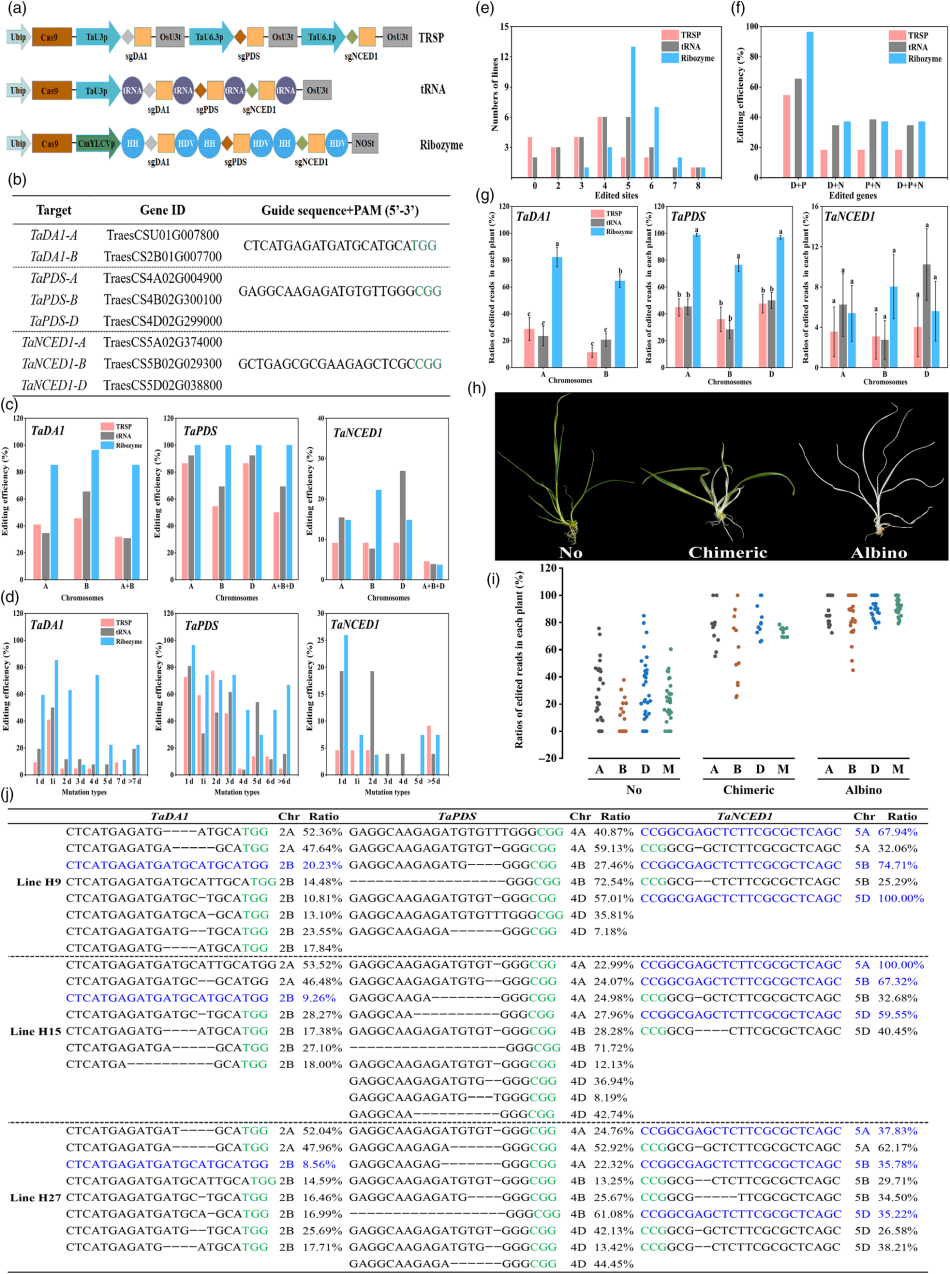
Multiplex genome editing mediated by CRISPR/Cas9 system in common wheat. (a) Illustration of vectors for three distinct multiplex CRISPR/Cas9 systems used in this study. The protospacers and sgRNA scaffolds are indicated by diamonds and orange, respectively. (b) Target gene IDs and sequences of the target sites. The PAM is labelled in green. (c) Editing efficiencies of homoeologous genes at different subgenomes in three multiplex editing systems. A + B, plants with genes edited at both A and B chromosomes. A + B + D, plants with genes simultaneously edited at A, B and D chromosomes. (d) Editing efficiencies of mutation types. The number of nucleotide insertion (i) and deletion (d) is indicated for each mutation type. (e) Number of lines with different numbers of edited sites. (f) Multiplex gene editing efficiencies in three editing systems: D, *TaDA1*; P, *TaPDS*; and N, *TaNCED1*. (g) Ratios of edited reads for homoeologous genes at different subgenomes in each plant. Data are presented as the mean ± SE. *n* = 22, 26, 27 for TRSP, tRNA and ribozyme systems, respectively. Different letters above the columns indicate groups with significant differences (ANOVA, *P* < 0.05). (h) Representative lines with no, chimeric and albino phenotype with *TaPDS* editing. (i) Ratios of edited reads for *TaPDS* at different chromosomes and their means in plants with no, chimeric and albino phenotype. M, mean value for ratios of *TaPDS‐A*, *TaPDS‐B* and *TaPDS‐D* in each plant. Each dot represents the ratios of edited reads in a single plant. A total of 31, 12 and 32 T0 transgenic plants were collected with no, chimeric and albino phenotype, respectively, and used in this analysis. (j) Examples of genotyping results for three T0 mutants generated by the ribozyme system. The alleles for each target gene were quantified by Hi‐TOM NGS. The PAM is labelled in green, and the wild type alleles are shown in blue. Chr, chromosome.

A total of 22, 26 and 27 T0 plants were generated from the transformed calli of TRSP, tRNA and ribozyme systems, respectively. The genotype of each plant was characterized by Hi‐TOM sequencing of the PCR amplicons with primers flanking each target site (Liu *et al*., 2019). The editing efficiency of individual genes was first analysed. Edits of *TaDA1‐A* and *TaDA1‐B* were detected in all three systems, and the ribozyme system was most effective (Figure 1c). The superior editing ability of the ribozyme system was also observed at *TaPDS* where mutations could cause albino phenotype. Impressively, 22 out of 27 plants showed albino phenotype in the ribozyme system, whereas only 5 plants displayed albino phenotype in either the TRSP or tRNA system. Sequencing results supported the observation as the ribozyme system achieved the highest efficiency, up to 100.00% (Figure 1c). Although fewer plants showed albino phenotype, the editing efficiencies in TRSP and tRNA systems still reached to 86.36% and 92.31%, respectively. For *TaNCED1*, all three vectors exhibited low activity, and the tRNA and ribozyme systems resulted in higher gene editing rates than the TRSP system (Figure 1c). The three systems showed similar mutation profiles for individual genes where small deletions and 1bp insertions predominated (Figure 1d).

The ability to target multiple genomic sites was further analysed for three systems. Compared with the TRSP system, more plants with over 4 edited sites were identified in the tRNA and ribozyme systems (Figure 1e), and the ribozyme system generated the highest simultaneous editing rates. The efficiencies of simultaneous editing in three genes in the tRNA and ribozyme systems were 34.62% and 37.04%, respectively, which were about twofold higher than that in the TRSP system (Figure 1f). Thus, the tRNA and ribozyme systems are more effective than TRSP, and the ribozyme system appeared to be most robust. The high editing efficiency of the ribozyme system might partly result from the use of the Pol II promoter, CmYLCV, for sgRNA expression.

The phenotype caused by gene editing depends on the genotype in individual plants. Therefore, we investigated the ratio of mutated reads at each target site in each plant through Hi‐TOM sequencing (Liu *et al*., 2019). No significant differences were detected at all targeted sites between the TRSP and tRNA systems, but the ratios of edited reads were significantly increased in the ribozyme system except for *TaNCED1* (Figure 1g). Ratios of edited reads in the ribozyme system were about threefold higher for *TaDA1* and twofold higher for *TaPDS* than those in the TRSP and tRNA systems, respectively. The results suggested that the ribozyme system greatly decreased the proportions of unedited reads at multiplex chromosomes and therefore increased the probability of the loss‐of‐function phenotype in T0 generation. This might explain the discrepancy between the high editing efficiency and less albino phenotype caused by *TaPDS* mutation in the TRSP and tRNA systems. Although over 86.00% of the plants carried edited *TaPDS* in the TRSP and tRNA systems, the editing ratios in most plants were not enough to display albino phenotype. To further quantify the relationship between ratios of the edited reads and observable phenotype, we compared the ratios of edited reads for *TaPDS‐A*, *TaPDS‐B* and *TaPDS‐D* among three groups of plants as no albino, chimeric and albino phenotype, respectively (Figure 1h). Positive correlation between the phenotype and editing ratio was observed (Figure 1i). The lowest average ratio for plants with albino phenotype was 80.59%, indicating an editing threshold for displaying loss‐of‐function phenotype. Wild type alleles were not detected at *TaPDS* in 6 lines of the ribozyme system despite different levels of chimerism (Figure 1j). These results collectively revealed that the phenotype caused by targeted mutagenesis occurred only when the ratios of edited homoeologous genes achieved a higher level at all homoeologous chromosomes simultaneously.

In summary, we compared three multiplex CRISPR/Cas9 systems for simultaneous genome editing at 8 target sites in common wheat. The tRNA and ribozyme systems were more effective than the TRSP system in multiplex genome editing. Furthermore, the ribozyme system could significantly increase the ratios of edited homoeologous genes at multiplex chromosomes in individual plants and therefore generated more plants with loss‐of‐function phenotypes. The ribozyme system established in our study would greatly aid fundamental and translational research in wheat.

## Conflicts of interest

The authors declare no conflicts of interest.

## Author contributions

J.L. and S.Z. constructed the vectors, analysed the data and wrote the manuscript. R.Z. designed the sgRNAs and performed Hi‐TOM sequencing. G.S. and W.L. collected samples and extracted DNA. J.G. performed the wheat transformation. Y.Q. analysed the data and revised the manuscript. Y.L. and G.L supervised the project.
